# The Development and Role of Capmatinib in the Treatment of MET-Dysregulated Non-Small Cell Lung Cancer—A Narrative Review

**DOI:** 10.3390/cancers15143561

**Published:** 2023-07-10

**Authors:** Robert Hsu, David J. Benjamin, Misako Nagasaka

**Affiliations:** 1 Division of Medical Oncology, Department of Internal Medicine, Norris Comprehensive Cancer Center, University of Southern California, Los Angeles, CA 90033, USA; robert.hsu@med.usc.edu; 2 Hoag Family Cancer Institute, Newport Beach, CA 92663, USA; david.benjamin@hoag.org; 3 Division of Hematology and Oncology, Department of Medicine, Chao Family Comprehensive Cancer Center, University of California Irvine School of Medicine, Orange, CA 92868, USA

**Keywords:** NSCLC, MET dysregulation, capmatinib, tyrosine kinase inhibitor, detection

## Abstract

**Simple Summary:**

In this narrative review, we discuss the development of capmatinib, a reversible *MET* tyrosine kinase inhibitor that received approval for advanced non-small cell lung cancer (NSCLC) harboring *MET* exon 14 skipping mutation. Capmatinib was first discovered in 2011 and has been shown to have promising antitumor activity. Early-phase trials identified a recommended dose of 400 mg twice daily in tablet formulation. The GEOMETRY mono-1 trial showed efficacy in *MET* exon 14 skipping mutation, leading to FDA approval for capmatinib. Currently, ongoing clinical trials evaluating combination therapy with capmatinib, including amivantamab, trametinib, and immunotherapy, are being conducted to improve efficacy and broaden indications of capmatinib with new drug agents such as antibody–drug conjugates being developed to treat *MET* dysregulated NSCLC.

**Abstract:**

Non-small cell lung cancer (NSCLC) is a leading cause of death, but over the past decade, there has been tremendous progress in the field with new targeted therapies. The mesenchymal–epithelial transition factor (*MET*) proto-oncogene has been implicated in multiple solid tumors, including NSCLC, and dysregulation in NSCLC from *MET* can present most notably as *MET* exon 14 skipping mutation and amplification. From this, *MET* tyrosine kinase inhibitors (TKIs) have been developed to treat this dysregulation despite challenges with efficacy and reliable biomarkers. Capmatinib is a Type Ib *MET* TKI first discovered in 2011 and was FDA approved in August 2022 for advanced NSCLC with *MET* exon 14 skipping mutation. In this narrative review, we discuss preclinical and early-phase studies that led to the GEOMETRY mono-1 study, which showed beneficial efficacy in *MET* exon 14 skipping mutations, leading to FDA approval of capmatinib along with Foundation One CDx assay as its companion diagnostic assay. Current and future directions of capmatinib are focused on improving the efficacy, overcoming the resistance of capmatinib, and finding approaches for new indications of capmatinib such as acquired *MET* amplification from epidermal growth factor receptor (*EGFR*) TKI resistance. Clinical trials now involve combination therapy with capmatinib, including amivantamab, trametinib, and immunotherapy. Furthermore, new drug agents, particularly antibody–drug conjugates, are being developed to help treat patients with acquired resistance from capmatinib and other TKIs.

## 1. Introduction

Non-small cell lung cancer (NSCLC) is a leading cause of death, accounting for an estimated 1.8 million deaths according to GLOBOCAN in 2020 [1]. Over the past decade, there has been tremendous progress in the discovery and development of targeted therapies for *EGFR*; *KRAS* G12C; *BRAF* V600E mutations; *ALK*, *ROS1*; *RET* gene rearrangements; *MET* alterations, including *MET* exon 14 skipping mutations, *ERBB2* (HER2) mutations, and *NTRK* 1/2/3 gene mutations [2,3,4,5,6,7,8,9,10]. This has led to the personalization of medicine in NSCLC.

The mesenchymal–epithelial transition factor (*MET*) gene is located in human chromosome 7 (7q21–q31), comprising 21 exons and 21 introns, and encodes a protein that is approximately 120 kDa in size. The ligand for MET is hepatocyte growth factor (HGF), which is a soluble cytokine and is synthesized by mesenchymal cells, fibroblasts, and smooth muscle cells [11]. HGF will bind to MET, and this will trigger the autophosphorylation of Tyr-1234 and Tyr-1235 in the intracellular tyrosine kinase domain, which then undergoes further autophosphorylation of Tyr-1340 and Tyr-1356 in the C-terminal docking site [11,12]. This then facilitates the recruitment of intracellular effector molecules such as GRB2, SRC, PIK3, and GAB1, leading to the activation of downstream pathways. Normally, MET/HGF signaling pathway mediates embryogenesis, tissue regeneration, wound healing, and the formation of nerves and muscles [11,12,13].

**Figure 1:**
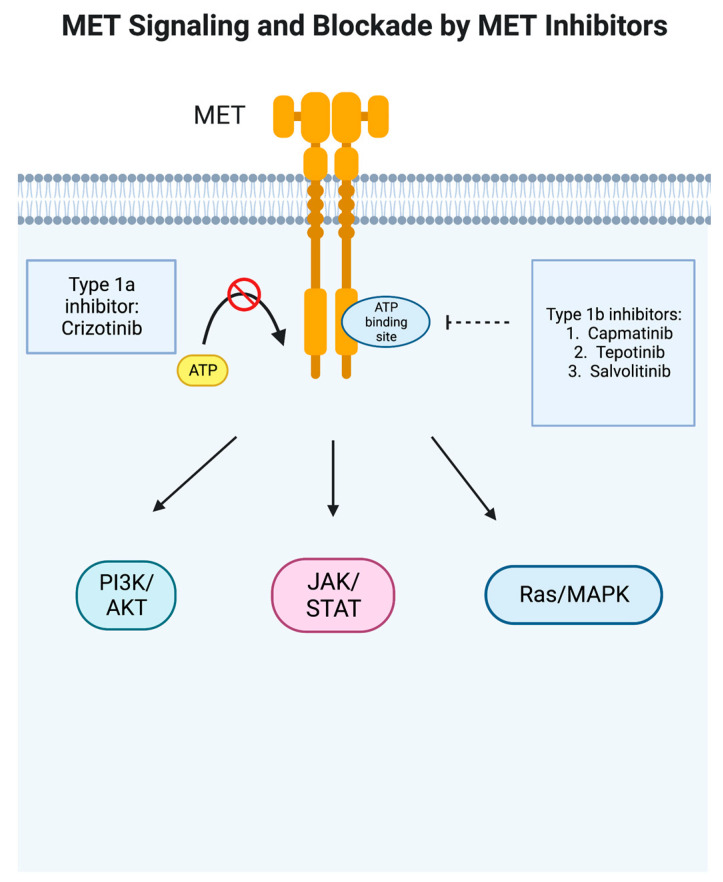
MET signaling pathway and blockade by MET inhibitors. In cancer, the *MET* proto-oncogene is abnormally activated and stimulates other signaling pathways in tumor cells, notably PI3K/AKT, JAK/STAT, Ras/MAPK, SRC, and Wnt/beta-catenin [11]. Type 1a inhibitor crizotinib blocks ATP binding to prevent the phosphorylation of the receptor, whereas type 1b inhibitors such as capmatinib are more specific and bind to a pocket adjacent to the ATP binding site. This figure was generated by BioRender.

In cancer, the *MET* proto-oncogene is abnormally activated and stimulates other signaling pathways in tumor cells, notably PI3K/AKT, JAK/STAT, Ras/MAPK, SRC, and Wnt/beta-catenin [11] (Figure 1). *MET* overexpression can be found in inflammation and hypoxia, leading to proliferation and migration, and is seen in a large variety of cancer types, including epithelial, mesenchymal, and hematological malignancies [14]. In NSCLC, it has been shown to be overexpressed in 35–72% of cases [14]. High levels of *MET* expression have been found to correlate with early disease recurrence [15]. *MET* dysregulation in NSCLC can present in a variety of ways—gene overexpression; HGF expression that can cause ligand-induced activation, leading to sustained or altered signaling; gene amplification, which can lead to overexpression and reduce the requirement for ligand activation, leading to sustained or altered signaling of the *MET* receptor; gene rearrangement, which may reduce or remove the requirement for ligand activation, leading to sustained altered signaling properties of the *MET* receptor; and downstream MET signaling alterations [11,12,15]. Notably, cigarette smoking can upregulate c-MET and the downstream Akt pathway [16]. It also affects the sensitivity of *EGFR* TKIs as cigarette smoke attenuates the AMP-activated protein-kinase (AMPK)-dependent inhibition of mTOR which then decreases the sensitivity of NSCLC cells with wild-type *EGFR* to TKI and thereby represses the expression of liver kinase B1 (LKB1) [17]. Finally, *MET* dysregulation can occur via gene mutation, most notably the *MET* exon 14 skipping mutation seen in about 3–4% of adenocarcinoma and 2% of squamous cell carcinoma but in higher frequencies in adenosquamous carcinoma (6%) and pulmonary sarcomatoid carcinoma (9–22%) [15,18]. 

*MET* exon 14 skipping mutations are processes in which the 47-amino-acid juxtamembrane domain is deleted, altered, or disrupted by intronic regions surrounding exon 14, leading to fusion in mature mRNA between exon 13 and exon 15 [19,20]. *MET* exon 14 skipping mutations have been shown to be exclusive from other driver mutations but coexist with other *MET* amplification or copy number gains [21]. Meanwhile, the amplification of the *MET* gene, which is defined as a gain in copy number (GCN), has been seen both de novo and as an acquired resistance mechanism [22]. *MET* amplification is seen in *EGFR*-acquired resistance and can occur with or without the loss of T790M [23]. In the analysis of resistance mechanisms in the AURA 3 study (*n* = 78), *MET* amplification was seen in (14/78,18%) of samples, *EGFR* C797S (14/78,18%) of cases, and 15 patients having >1 resistance-related genomic alteration [23,24]. *MET* amplification is also considered an acquired resistance mechanism of *ALK* inhibitors, as *MET* amplification has been observed in about 15% of next-generation *ALK* inhibitor resistance [25]. Both *MET* exon 14 skipping mutations and *MET* high-level amplification have been shown to portend poor prognosis [21]. Without the use of *MET* inhibitors, a retrospective study by Awad et al. showed that the median OS was 8.1 months [26]. *MET* exon 14 skipping mutations are seen more frequently in females than in males, and the median age of *MET* exon 14 skip mutation patients ranged from 71.4 to 76.7 years [6,18]. Compared with other driver mutations, *MET* exon 14 skip mutation patients tend to be smokers, with only about 36% being never smokers in a previous retrospective analysis [27].

*MET* tyrosine kinase inhibitors have been developed to treat *MET*-dysregulated NSCLC, classified as Type I, Type II, and Type III inhibitors. Type I inhibitors compete with ATP for the binding of the ATP-binding pocket of the active conformation of *MET*. Specifically, Type Ia inhibitors such as crizotinib interact with the Y1230 residue in the hinge region and are dependent on binding with the G1163 residue [28,29]. Type Ib inhibitors such as capmatinib, tepotinib, and savolitinib also connect with the Y1230 residue but are not dependent on G1163 binding [28,30,31,32]. Meanwhile, Type II inhibitors, which include cabozantinib, meresitinib, and gleasatanib, bind the ATP pocket in an inactive state [32,33,34,35]. Type III inhibitors bind to allosteric sites different from the ATP site and are not competitive; tivantinib has been studied in NSCLC but was not found to show any benefit in interim analysis and therefore was discontinued [32,34,36].

This review specifically focuses on capmatinib (INC280), which received U.S. Food and Drug Administration (FDA) approval for *MET* exon 14 skip mutations in metastatic NSCLC on 10 August 2022 and by the European Medicines Agency (EMA) on 20 June 2022 specifically for those patients who have received immunotherapy or platinum-based chemotherapy or both [37,38]. Herein, we review clinical development trials involving capmatinib, notably the GEOMETRY mono-1 study, which led to FDA approval and the companion diagnostic assay for the detection of MET exon 14 skipping mutations.

## 2. Crizotinib

Prior to capmatinib, crizotinib was the first *MET* TKI to show efficacy in *MET* exon 14 skipping mutation in advanced NSCLC. The PROFILE 1001 trial showed an overall response rate (ORR) of 32% (95% CI 21–45) among 65 response-evaluable patients, with a median duration of response (DOR) of 9.1 months (95% CI 6.4–12.7) and a progression-free survival (PFS) rate of 7.3 months (95% CI 5.4–9.1), with two additional Phase II crizotinib trials showing ORR of around 30% [39,40,41]. However, crizotinib confers resistance to G1163R mutation not seen in *MET* Type Ib TKIs such as capmatinib, and thus treatment for *MET* dysregulation has shifted towards *MET* Type Ib TKIs [42]. Currently, crizotinib is approved for *ALK*- and *ROS1*-positive advanced NSCLC by the FDA and EMA [43,44].

## 3. Preclinical Studies

Capmatinib was first reported in 2011 by Liu et al., who showed that in both in vivo and in vitro mice studies using human cell lines, capmatinib had a 10,000-fold selectivity for c-met over a large panel of human kinase [45]. They showed that capmatinib can block the c-MET phosphorylation and activation of downstream targets, including HGF. They further showed that activated c-met upregulates cancer-promoting EGFR and HER-3 pathways [45]. Baltschukat et al. further investigated capmatinib in NSCLC [46]. They investigated the affinity of capmatinib in a set of 442 kinases and demonstrated a selectivity in *MET* of over 1000 fold [46]. Furthermore, they demonstrated that capmatinib is highly selective to Y1230 and D1228 and observed resistance when using cell lines bearing mutations to Y1230 and D1228 [46]. *MET* amplification and HGF expression in vitro were also associated with capmatinib sensitivity in vitro [46].

## 4. Pharmacodynamics/Pharmacokinetics

**Figure 2:**
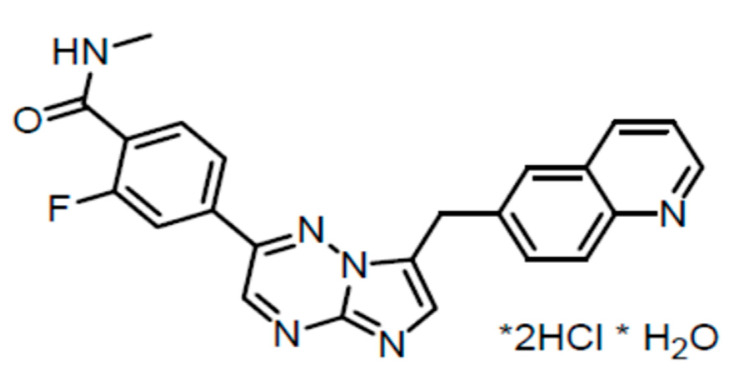
Chemical structure of capmatinib; the asterisk (*) represents the chiral carbons that are part of the chemical structure.. The chemical name for capmatinib is 2-Fluoro-N-methyl-4-[7-(quinolin-6-ylmethyl)imidazo[1,2 b][1,2,4]triazin-2-yl]benzamide—hydrogen chloride—water (1/2/1). The molecular formula for capmatinib hydrochloride is C_23_H_21_Cl_2_FN_6_O_2_ [38].

Capmatinib is a selective Type Ib ATP-competitive tyrosine kinase inhibitor targeting *MET.* Capmatinib has an average IC_50_ value of 0.13 nM and a cell-based IC_50_ of 0.3–0.7 nM in lung cancer cell lines [28,46] (Figure 2). Capmatinib has linear pharmacokinetics, with exposure increasing approximately dose-proportionally over a dose range of 200–400 mg. It is rapidly absorbed, with peak plasma concentration (C_max_) obtained about 1–2 h after a 400 mg dose is given. There is similar absorption when taken with and without food. The effective elimination half-life is 6.5 h. The plasma protein binding is 96% [38,47].

Capmatinib is metabolized by CYP3A4 and aldehyde oxidase. In a single oral dose, 78% of total radioactivity was recovered in feces with 42% as unchanged and 22% recovered in urine. There are no specific significant effects on the pharmacokinetic parameters of capmatinib identified in the following covariates assessed: age, sex, race, mild-to-moderate renal impairment, and hepatic impairment [38,47].

In drug interaction studies, coadministration with itraconazole, a strong CYP3A inhibitor, increased capmatinib’s area under the curve (AUC_0-INF_) by 42%, with no change in C_max_. Coadministration with rifampicin, a strong CYP3A inducer, decreased capmatinib AUC_0-INF_ by 67% and decreased C_max_ by 56%. Coadministration with protein pump inhibitors (rabeprazole) decreased capmatinib by AUC_0-INF_ 25% and decreased C_max_ by 38%. Coadministration with rosuvastatin, a BRCP substrate, increased rosuvastatin AUC_0-INF_ by 108% and increased C_max_ by 204% [38,47].

## 5. Phase I Clinical Trials

Multiple open-label, multicenter, Phase I studies in advanced solid tumors have evaluated capmatinib. A Phase I study comprising 44 adult Japanese patients, including 15 NSCLC patients, found that the highest studied dose determined to be safe was 400 mg administered orally (po) twice a day (b.i.d.) as a tablet. The median duration of treatment exposure was 7 weeks (range 0.4–32.3 weeks), with disease progression being the primary reason for the discontinuation occurring in 38 patients (86.4%). There were two drug-limiting toxicities (DLTs), which consisted of Grade 2 suicidal ideation in a patient taking 600 mg po b.i.d. and Grade 3 depression in a patient taking 400 mg po b.i.d. [48]. Another global Phase I study, comprising 38 patients primarily with gastrointestinal cancers, had a recommended Phase II dose (R2PD) of 600 mg po b.i.d. in a capsule formulation and 400 mg po b.i.d. in a tablet formulation. The most frequent Grade 3 or 4 adverse events were an increase in levels of blood bilirubin (11%), fatigue (8%), and AST increase (8%) [49].

Schuler et al. investigated 55 patients with advanced *MET*-dysregulated NSCLC, which included 40 patients with prior systemic therapies. All patients discontinued treatment, mostly due to disease progression (69.1%), with a median duration of 10.4 weeks. While the overall response rate (ORR) by RECIST for the entire cohort was 20%, *MET* with a gene copy number ≥6 had an ORR of 47%, with median progression-free survival (PFS) of 9.3 months, and all patients with *MET* exon 14 skip mutations had a response. The most common toxicities were nausea (42%), peripheral edema (33%), and vomiting (31%) [50]. Another Phase Ib/II study involving capmatinib investigated *EGFR*-mutated, *MET-*dysregulated NSCLC in combination with gefitinib, an *EGFR* TKI, in patients with acquired *EGFR* TKI resistance. The ORR across the cohort was 27%, with a 47% ORR in patients with a *MET* copy number ≥6. The drug was relatively well tolerated, with the most common Grade 3–4 adverse event being increased amylase and lipase levels (6% in both). The R2PD was capmatinib 400 mg po b.i.d. plus gefitinib 250 mg po daily [51] (Table 1).

## 6. GEOMETRY Mono-1 Trial

The GEOMETRY mono-1 trial was a multicohort Phase II study in patients with *MET*-dysregulated advanced NSCLC. The patients were either in Stage IIIB or IV NSCLC, had no *EGFR* mutation, and were negative for *ALK* rearrangement. All subjects took capmatinib 400 mg po b.i.d. A total of 364 patients were enrolled, with 97 having a *MET* exon 14 skipping mutation and 210 having *MET* amplification. There were seven cohorts to the study: In previously treated patients (1–2 lines of therapy), Cohort 1 consisted of *MET* amplification with (a) GCN ≥ 10 (*n* = 69) or (b) GCN 6–9 (*n* = 42); Cohort 2 consisted of *MET* amplification with GCN 4–5 (*n* = 54); Cohort 3 consisted of *MET* amplification with GCN < 4 (*n* = 30); Cohort 4 consisted of *MET* exon 14 skipping mutation with any GCN (*n* = 69); and Cohort 6 consisted of *MET* amplification with GCN > 10 (*n* = 3) or *MET* exon 14 skipping mutation with any GCN (*n* = 31) who had received one line of therapy (*n* = 34). In the untreated group, Cohort 5a consisted of *MET* amplification with GCN ≥ 10 (*n* = 15); Cohort 5b consisted of *MET* exon 14 skipping mutation with any GCN *(n* = 28); and Cohort 7 consisted of treatment-naïve *MET* exon 14 skipping mutation with any GCN (*n* = 23). *MET* exon 14 skipping mutation patients had a slightly higher median age (71 years) than patients with *MET* amplification (60–70 years) on diagnosis. Patients with *MET* exon 14 skipping mutation were more likely to be women and to have never smoked [6].

Among patients with *MET* exon 14 skip mutations, ORR was seen in 41% (95% CI 29–53) of 69 previously treated patients and 68% (95% CI 48–84) of 28 previously untreated patients. The median duration of response (DOR) was 9.7 months (95% CI 5.6–13.0) among the treated patients and 12.6 months (95% CI 5.6—not reached) in previously untreated patients. Most patients (82% in treated and 68% in untreated) had a response at the first tumor evaluation following the start of capmatinib therapy. The median PFS was 5.4 months (95% CI 4.2–7.0) in previously treated patients and 12.4 months (95% CI 8.2—not reached) in previously untreated patients. Notably, 12 of 13 patients with exon 14 skipping mutations who had brain metastasis had intracranial disease control. The primary reason for discontinuation was progressive disease (58% in previously treated patients and 46% in untreated patients) [6].

In patients with GCN < 10, the cohorts were closed due to futility, as PFS for GCN 6–9 and 4 or 5 was only 2.7 months. In GCN ≥ 10, there was activity; the ORR was 29% (95% CI 19–41) in previously treated patients and 40% (95% CI 16–68) in previously untreated patients, but this fell below the predefined clinical efficacy. The median DOR was 8.3 months (95% CI 4.2–15.4) in treated patients and 7.5 months (95% CI 2.6–14.3) in untreated patients. The median PFS was 4.1 months (95% CI 2.9–4.8) in treated patients and 4.2 months (95% CI 1.4–6.9) in untreated patients [6] (Table 2).

Across all cohorts, the most reported adverse events were peripheral edema, nausea, and vomiting. Overall, 67% of patients had adverse events of Grade 3 or 4; the most frequent of these were peripheral edema, nausea, vomiting, and increased blood creatinine level. Treatment-related adverse events led to the discontinuation of treatment in 39 patients (11%), with treatment-related peripheral edema leading to discontinuation in 6 patients (2%) [6].

The post hoc analysis involving 69 *MET* exon 14 skipping mutation patients that focused on 19 patients in the cohort who had previously received immunotherapy (IO) showed ORR 57.9% (*n* = 11/19; 95% CI 33.5–79.5%), with a median DOR of 11.2 months (95% CI 3.35—not reached). Safety findings were similar, according to which capmatinib showed efficacy irrespective of prior treatment with IO and was also well tolerated in post-IO patients [52]. Moreover, capmatinib was associated with clinically meaningful improvements in cough and preserved the quality of life in patient-reported surveys [53]. There was also a subgroup analysis on 45 Japanese patients, which showed an ORR of 36% (95% 10.9–69.2) and good tolerability [54].

A recent real-world analysis was carried out that investigated *MET* exon 14 skipping mutation and brain metastasis patients; of the 68 patients that fit the criteria, the real-world response rate was 90.9%, with 87.3% intracranial response along with a median PFS rate of 14.1 months [55]. Another real-world retrospective study examined 81 cases of NSCLC with advanced NSCLC and *MET* exon 14 skipping mutation who were treated with capmatinib from March 2019 to December 2021 [56]. The ORR to capmatinib was 58% (95% CI 47–69), including 68% (95% CI 50–82) for treatment-naïve and 50% (95% CI 35–65) for pretreated patients. The median PFS was 9.5 months (95% CI 4.7–14.3), and the median OS was 18.2 months (95% CI 13.2) for the entire cohort, including a median PFS of 10.6 months (95% CI 5.5–15.7) for untreated patients [56].

Thus, the GEOMETRY mono-1 trial evaluated *MET*-dysregulated, advanced NSCLC, with promising ORR and PFS seen in *MET* exon 14 skip mutations, though the results showed a lack of effect in *MET* GCN < 10, leading to FDA and EMA approval for capmatinib only in advanced NSCLC with *MET* exon 14 skipping mutations. Subsequent real-world data have shown response to capmatinib among patients with *MET* exon 14 skipping mutations, with IO exposure and brain metastasis [52,56].

## 7. Tepotinib and Savolitinib

Two other MET selective Type Ib inhibitors have been investigated in *MET* alterations, namely tepotinib and savolitinib [30,31]. Tepotinib received accelerated approval from the FDA for *MET* exon 14 skipping mutations in advanced NSCLC after the open-label Phase II VISION study [31]. It also received approval from the EMA for those with advanced NSCLC *MET* exon 14 skipping mutations who require systemic therapy following immunotherapy and/or platinum-based therapy [57]. In this study, 152 patients with *MET* exon 14 skipping mutations were followed, and the ORR was 46% (95% CI 36–57), including 44.2% (95% CI 29.1–60.1) in untreated patients and 48.2 (95% CI 34.7–62.0) in previously treated patients. The median DOR was 11.1 months (95% CI 7.2—not reached), and the PFS was 8.5 months (95% CI 5.1–11.0) [31]. There were 11 patients with brain metastasis in the study, with a median PFS of 10.9 months (95% CI 8.0—not reached) [31].

Meanwhile, a Phase II, single-arm, open-label study in China involved 84 patients with *MET* exon 14 skipping mutations who had positive pulmonary sarcomatoid carcinoma or other NSCLC subtypes and received savolitinib [30]. The ORR was 42.9% (95% CI 31.1–55.3) [20] (Table 3). Savolitinib received conditional approval in China in 2021 for the treatment of metastatic NSCLC with *MET* exon 14 skipping mutations in patients who have progressed after or who are unable to tolerate platinum-based chemotherapy [58].

## 8. Companion Diagnostic Assay

One of the challenges in the success of finding successful *MET*-targeted therapies has been finding a reliable biomarker. For example, in previous studies where *MET* GCN ≥ 6, the ORR outcomes ranged from 16% to 67%, while for immunohistochemistry (IHC) 2+ and 3+, the ORR outcomes ranged from 14% to 68% [6,39,40,41,50,51,59]. Another way to assess *MET* overexpression has been the *MET*/chromosome 7 centromere (CEP7) ratio, in which ORR outcomes range from 33% to 67% [51,59] (Table 4).

Some thoughts as to the lack of reliability in *MET* amplification have been that gene copy number gains can occur through both polysomy and amplification and thus the gene copy number could be a result of polysomy, not true amplification [59,60,61]. Another possible problem has been the use of NGS-based assays with a control group using CEP7 [59,61]. A previous study has shown that a *MET*/CEP7 ratio >5 is reliable for *MET* inhibitor response, but the issue is that many below this ratio have other oncogenes and may not be truly *MET*-addicted cases [61]. Guo et al. demonstrated that MET expression via mass spectrometry, IHC, and H-score ≥ 200 had significantly improved PFS but saw no association based on copy number [62].

Another challenging aspect of finding a reliable companion diagnostic assay has been the discrepancy between circulating tumor DNA (ctDNA) and tumor next-generation sequencing (NGS) testing. Ikeda et al. studied the ctDNA of 438 patients, and among the 31 patients with *MET* alterations, only 2 of the 18 patients who also received tissue testing were found to have *MET* alterations in the tissue [63]. Another study involving paired plasma and tissue samples in advanced NSCLC patients showed 77.6% concordance between tissue and plasma NGS; 26% of the cohort who received both ctDNA and tissue testing had *MET* alterations on ctDNA testing, but only 17.8% of the 26% total also had *MET* alterations on tissue testing [64]. Overall, when compared to tumor NGS testing, ctDNA had 67.7% sensitivity and 88.8% specificity in pretreated patients, whereas in treated patients, it revealed a sensitivity of 68.4% but only a specificity of 16.7% [64]. Yet, *MET* alterations have been found in both circulating-free DNA (cfDNA) and circulating tumor cells (CTCs) both at diagnosis and at resistance to *EGFR* TKIs [65]. Moreover, Peng et al. examined 48 paired samples and showed a 92.4% concordance between the absolute copy number variant > 6 and the NGS detection of *MET* amplification in tumor tissue [66]. This all has significant ramifications clinically when it comes to making sure *MET* dysregulation is captured on diagnosis but then also on acquired resistance because sometimes patients may not have adequate tissue for testing, which limits them only to liquid biopsy testing, or clinicians may choose to only perform liquid biopsy testing upon the progression of the disease. Thus, finding a trustable biomarker, whether it is a specific *MET* GCN or *MET*/CEP7 ratio threshold that can be used in both tissue testing and ctDNA testing, will go a long way towards determining which *MET* amplification patients would benefit from capmatinib and other *MET*-targeted agents and to ensure that as many *MET* exon 14 skipping mutations are detected as possible.

In *MET* exon 14 skipping mutations, there is also some variability in the ORR, with ranges from 32% to 64%, though these studies do originate from patients on different lines of therapy and different *MET* TKI inhibitors [30,31,41]. However, in the GEOMETRY mono-1 trial, a clinical bridging study was carried out to show analytical and clinical agreement between the enrollment assay and the Foundation One CDx assay [59,67]. The Foundation One CDx assay, developed by Foundation Medicine in collaboration with Novartis, is performed at Foundation Medicine Inc. using DNA isolated from fresh-frozen paraffin-embedded (FFPE) tumor tissue specimens. In previously treated patients, the positive percent agreement (PPA) was 96.8%, the negative percent agreement (NPA) was 100%, and the overall agreement (OA) was 100%. In untreated patients, the PPA, NPA, and OA were all 100%. This led to the FDA approval of the Foundation One CDx assay as the only assay associated with a *MET* inhibitor [59,67].

## 9. Toxicities

In the GEOMETRY mono-1 trial, across all cohorts, the most reported adverse events were peripheral edema, nausea, and vomiting. Notably, 67% of patients had adverse events of Grade 3 or 4; the most frequent of these were peripheral edema, nausea, vomiting, and increased blood creatinine level. Treatment-related adverse events led to the discontinuation of treatment in 39 patients (11%), with treatment-related peripheral edema leading to discontinuation in 6 patients (2%) [6].

In the VISION study, 28% of patients had Grade 3–4 adverse events, with peripheral edema (7%) being the greatest [31]. Other Grade 3–4 adverse events with greater than 1% incidence included increased amylase (3%), increased lipase (3%), pleural effusion (3%), increased ALT (3%), increased AST (2%), and general edema (3%) [31]. Meanwhile, in the study involving savolitinib, treatment-related adverse events occurred in 46% of the patients, with increased aspartate aminotransferase (*n* = 9), alanine aminotransferase (*n* = 7), and peripheral edema (*n* = 6) being the most common serious adverse side effect. There was one death in the study due to tumor lysis syndrome, likely treatment-related [30] (Table 5).

## 10. Discussion and Future Directions

Although capmatinib has been approved by both the FDA and EMA, there has not been a Phase III trial comparing capmatinib versus chemotherapy and immunotherapy in the first-line setting for *MET* exon 14 skipping mutations despite the National Comprehensive Cancer Network (NCCN) recommending capmatinib as first-line therapy in advanced NSCLC with *MET* exon 14 skipping mutations [68]. In pretreated populations, the GEOMETRY-III (NCT04427072) trial is a study that involves approximately 90 previously treated advanced NSCLC patients harboring *MET* exon 14 skipping mutation and compares the efficacy of capmatinib with docetaxel [69]. Furthermore, capmatinib has been studied in 20 patients previously treated with a *MET* inhibitor, including 15 with *MET* exon 14 skipping mutation. The DCR was 80%. Notably, circulating tumor DNA analysis was carried out on these patients, and a secondary *MET* mutation was detected in four patients with *MET* D1228H and Y1230H, along with three patients having MAPK signaling alterations [70]. Furthermore, capmatinib and other Type Ib *MET* inhibitors have not been directly compared with Type Ia *MET* inhibitors.

Meanwhile, the challenge remains in finding reliable combinations to both improve the efficacy of capmatinib and broaden the indications of capmatinib use beyond MET exon 14 skipping mutations (Table 6). 

Within population subgroups, there are ongoing studies on capmatinib in Asia, which may give insight into its efficacy within specific Asian subgroup populations, including one in China (GEOMETRY-C study, NCT04677595) and one in India (NCT05110196). For early-stage NSCLC, the GEOMETRY-N (NCT04926831) study is a Phase II, two-cohort, two-stage study evaluating the efficacy and safety of neoadjuvant and adjuvant capmatinib therapy in improving the major pathological rate (MPR) and outcomes in patients with MET exon 14 skipping or high-level MET amplification NSCLC [71]. As there has been success with EGFR mutations and the use of osimertinib in an adjuvant setting with the ADUARA trial, it will be interesting to note the results of the major pathological response rate in this study [72].

Currently, there is a Phase I/Ib trial underway that investigates capmatinib and trametinib, a MEK inhibitor (NCT05435846), which may be of benefit to patients with progression on crizotinib. Meanwhile, there has not been much success with capmatinib in combination with immunotherapy due to limited activity and tolerability. A retrospective study at two academic institutions showed an ORR of 17% (95% CI 6–36) in MET exon 14 skip mutations receiving PD-L1 blockade [73]. A Phase II study (NCT04323436) looking at the efficacy and safety of capmatinib plus spartalizumab, a PD-1 monoclonal antibody, did not demonstrate significant antitumor benefit, with a high dose reduction/interruption (80.6%) and discontinuation rate (35.5%) [74]. Another Phase II randomized, open-label study (NCT04139317) evaluated the efficacy and safety of combination therapy with capmatinib and pembrolizumab versus pembrolizumab alone in first-line therapy among advanced NSCLC patients with PD-L1 tumor proportion score (TPS) ≥ 50% and no *EGFR* mutation or *ALK* rearrangements. However, the trial closed due to concerns from the drug sponsor of tolerability in patients [75]. Finally, there was another study that investigated the efficacy of capmatinib plus nivolumab or nazartinib (EGF816) plus nivolumab in previously treated NSCLC patients (NCT02323126). This study was also terminated due to low accrual, but in its primary endpoint of PFS at 6 months, capmatinib plus nivolumab showed a 68.9% (95% CI 48.85–85.7) PFS at 6 months in high cMet and 50.9% (95% CI 35.6–66.4) in low cMet (NCT02323126). However, there continue to be clinical trials, particularly with cabozantinib and atezolizumab (NCT03170960 and NCT04471428) targeting the *MET* pathway, as *MET* expression has been found to be implicated through its pathway with MET/HGF and is involved in the regulation of the inflamed tumor microenvironment, leading towards the upregulation of inhibitory molecules such as PD-L1 and the downregulation of immune stimulators such as CD137, CD252, and CD70 [76].

Another important role of capmatinib in the future is in patients with acquired *MET* amplification, as observed in about 15% of patients who received first-line osimertinib and in 12–22% of patients receiving second-line osimertinib [60]. As mentioned earlier, Wu et al. saw efficacy using capmatinib and gefitinib, and the TATTON trial, which incorporated osmertinib and savolitinib, showed ORR of 23–66% between the two arms of treatment [51,77]. The GEOMETRY-E study (NCT04816214) was a Phase III study involving osimertinib with capmatinib but recently closed due to a business decision, but a recent Phase II LUNG-MAP trial with SWOG (NCT05642572) recently opened that investigates capmatinib with osimertinib +/− ramucirumab in *EGFR* mutant, *MET*-amplified Stage IV or recurrent NSCLC. Meanwhile, NCT03040973 is a rollover study currently accruing in patients who were part of a Novartis-sponsored clinical trial to continue receiving capmatinib as a single agent or in combination with other treatments.

As with all TKIs, it will be important to note the recurring resistance mechanisms with capmatinib to aid with future directions. Previous studies have shown that in Type I MET TKIs, secondary mutations at residue Y1230 may cause resistance, as Type I MET TKIs do interact with Y1230, specifically Y1230C [42,78,79]. However, notably, D1228 mutations have also been seen in capmatinib and other Type I TKIs [42,46,79,80].

While switching to Type II MET TKIs has been believed to help overcome resistance to capmatinib, novel drugs that can bypass the *MET* signaling pathway may provide the answer for treatment in the post-capmatinib treatment setting [79]. Amivantamab, a bispecific, monoclonal antibody targeting *EGFR* and *MET* is a promising combination that can be considered in conjunction with capmatinib. In the CHYRSALIS study specifically involving patients with *MET* exon 14 skipping mutation whose disease had progressed or had declined standard-of-care therapy, the ORR was 21% (4/19) in patients with prior *MET* inhibitor therapy and 46% (5/11) in patients with no prior *MET* inhibitor therapy. The median DOR was not reached, and 67% (8/13) had DOR ≥ 6 months [81]. Meanwhile, in another cohort of patients with *EGFR* exon 19 deletion or L858R NSCLC who had progressed on an *EGFR* TKI, ORR with amivantamab and lazertinib, an *EGFR* inhibitor, was 36% (95% CI 23–51), and 39% had a DOR ≥ 6 months [82]. An ongoing clinical trial (NCT05488314) is currently underway that investigates the combination of amivantamab and capmatinib in advanced NSCLC with *MET* exon 14 skipping mutation or MET amplification and may provide a promising new combination. Another promising class of novel drugs includes antibody–drug conjugates (ADCs) in which the monoclonal antibody binds to a specific protein and can deliver a cytotoxic drug to its intended target [83]. telisotuzumab vedotin (Teliso-V) is an antibody–drug conjugate composed of a c-Met antibody (ABT-700) and a microtubule inhibitor (monomethyl auristatin E); the ongoing Phase II M14-239 LUMINOSITY trial (NCT03539536) showed a 52% ORR in patients with previously treated c-MET overexpressors with nonsquamous pathology and *EGFR* wild-type [84]. ABBV-400 is another ADC, which targets c-Met and topoisomerase-1, with an ongoing Phase I study (NCT05029882) involving c-Met overexpression in advanced solid tumors. In addition, a biparatopic MET x MET ADC REGN 5093-M114 has shown promising preclinical activity in both MET-overexpressed, TKI-naïve, EGFR-mutant NSCLC cells regardless of MET gene copy number as well as cell lines of EGFR-mutant NSCLC with PTEN loss or MET Y1230C mutation after the progression of prior osimertinib and savolitinib treatment [85]. A Phase I study (NCT04982224) is ongoing that involves the study of REGN5093-M114 in MET overexpression in advanced solid tumors.

Finally, it is worth noting the tolerability of capmatinib, as 67% of patients in the GEOMETRY mono-1 trial had a Grade 3 or 4 toxicity, and 42% of patients had serious adverse events [6]. The most frequent etiologies for Grade 3–4 toxicity include peripheral edema (9%), dyspnea (7%), fatigue (4%), and asthenia (4%), which all can severely impact the quality of life in patients [6]. While some of these side effects like peripheral edema can be controlled with supportive care, the toxicity profile of capmatinib merits further comparison with other standard-of-care options in a Phase III study and real-world prospective studies that evaluate side effects of capmatinib in clinical practice [86].

Thus, future directions in capmatinib and other combinations and novel agents in MET-dysregulated NSCLC will focus on the efficacy of these drugs, tolerability, and given the multiple new drugs, the sequence of these agents. 

## 11. Conclusions

The dysregulation of *MET* in NSCLC has proven challenging when it comes to finding therapeutic options given the lack of activity and reliability of biomarkers. Capmatinib, a Type Ib *MET* TKI that is not dependent on G1163, as crizotinib is, has proven to have efficacy, as shown in the GEOMETRY mono-1 study. Subsequent post hoc analyses have shown similar efficacy regardless of the prior treatment used and patient-reported improvement in quality of life. In addition, real-world analysis has shown similar efficacy with a promising intracranial response. The Foundation One CDx assay has been shown to be a reliable companion assay and remains the only FDA-approved assay for *MET*-targeted therapies. However, there have been no completed Phase III studies comparing capmatinib to first-line chemotherapy and immunotherapy or second-line chemotherapy. Furthermore, there was a notable percentage of Grade 3–4 toxicities. Future studies include investigations of capmatinib with *MEK* inhibition, combination therapy with amivantamab, and new classes of drugs, particularly ADCs. Capmatinib’s role in a perioperative setting in early-stage NSCLC may provide further treatment options for early stage patients with MET exon 14 skipping NSCLC, but the sequencing of these drugs and tolerability will be key factors, along with finding a more reliable biomarker.

## Figures and Tables

**Table 1:** Early-stage studies on capmatinib.

Publication	*n*	Indication	R2PD	ORR
Esaki et al. [48]	44 (15 NSCLC)	Advanced solid tumors	400 mg po bid	
Bang et al. [49]	38 (1 NSCLC)	Advanced solid tumors	600 mg po bid (capsule)/400 mg po bid (tablet)	
Schuler et al. [50]	55	Advanced NSCLC	600 mg po bid (capsule)/400 mg po bid (tablet)	47%
Wu et al. [51]	61 Phase Ib/100 Phase II	Advanced NSCLC in patients with acquired *EGFR* TKI resistance	400 mg po b.i.d. plus gefitinib 250 mg po daily	27% (47% in patients with MET GCN ≥ 6)

**Table 2:** Responses to capmatinib treatment relative to the cohort in GEOMETRY mono-1 trial (6).

Response	NSCLC with *MET* Exon 14 Skipping Mutation			NSCLC with *MET* Amplification			
Best Response—No (%)	Cohort 4 *n* = 69, any GCN with 1–2 Lines of Therapy	Cohort 5b *n* = 28, any GCN with No Previous Therapy	Cohort 1a *n* = 69, GCN ≥ 10 with 1–2 Lines of Therapy	Cohort 5a *n* = 15, GCN ≥ 10 with No Previous Therapy	Cohort 1b *n* = 42, GCN 6–9 with 1–2 Lines of Therapy	Cohort 2 *n* = 54, GCN 4 or 5 with 1–2 Lines of Therapy	Cohort 3 *n* = 30, GCN < 4 with 1–2 Lines of Therapy
Complete response	0	1 (4)	1 (1)	0	0	0	0
Partial Response	28 (41)	18 (64)	19 (28)	6 (40)	5 (12)	5 (9)	2 (7)
Stable disease	25 (36)	7 (25)	28 (41)	4 (27)	17 (40)	20 (37)	14 (47)
Incomplete response or nonprogressive disease	1 (1)	1 (4)	1 (1)	0	1 (2)	0	0
Unknown or could not be evaluated	9 (13)	0	8 (12)	1 (7)	4 (10)	8 (15)	8 (27)
Overall response							
No. of patients with overall response	28	19	20	6	5	5	2
Percent of patients (95% CI)	41 (29–53)	68 (48–84)	29 (19–41)	40 (16–68)	12 (4–26)	9 (3–20)	7 (1–22)
Disease control							
No. of patients with disease control	54	27	49	10	23	25	16
Percent of patients (95% CI)	78 (67–87)	96 (82–100)	71 (59–81)	67 (38–88)	55 (39–70)	46 (33–60)	53 (34–72)
Duration of Response							
No. of events/No. of patients with response	23/28	11/19	15/20	6/6	3/5	4/5	2/2
Median duration of response (95% CI)—mo	9.7 (5.6–13.0)	12.6 (5.6–NE)	8.3 (4.2–15.4)	7.5 (2.6–14.3)	24.9 (2.7–24.9)	9.7 (4.2–NE)	4.2 (4.2–4.2)
Progression-free survival							
Progression or death—No. of patients	60	17	58	15	34	50	22
Median progression-free survival (95% CI)—mo	5.4 (4.2–7.0)	12.4 (8.2–NE)	4.1 (2.9–4.8)	4.2 (1.4–6.9)	2.7 (1.4–3.1)	2.7 (1.4–4.1)	3.6 (2.2–4.2)

**Table 3:** Key trials involving *MET* selective Type 1b inhibitors.

	Capmatinib [6]	Tepotinib [31]	Savolitinib [30]
N (with MET exon 14 skipping mutation)	97	152 (99 evaluable)	84 (70 evaluable)
Overall response rate (%) (95% CI)	68% (48–84) in untreated patients (*n* = 28) and 41 (29–53) in previously treated patients (*n* = 69)	46 (36–57); 44.2% (29.1–60.1) in untreated patients (*n* = 43) and 48.2 (34.7–62.0) in previously treated patients (*n* = 56)	42.9 (31.1–53.3); 46.4 (27.5–66.1) in untreated patients (*n* = 28) and 40.5 (25.6–56.7) in previously treated patients (*n* = 42)
Duration of response mo (95% CI)	12.6 (5.6—NE) in untreated patients and 9.7 (5.6–13.0) in previously treated patients	11.1 (7.2—NE)	8.3 (5.3–16.6); 5.6 (4.1–9.6) in untreated patients and 9.7 (4.9—NE) In previously treated patients
Progression-free survival mo (95% CI)	12.4 (8.2—NE) in untreated patients and 5.4 (4.2–7.0) in previously treated patients	8.5 (5.1–11.0)	6.8 (4.2–9.6); 5.6 (4.1–9.6) in untreated patients and 6.9 (4.1–9.3) in previously treated patients

**Table 4:** Predictive biomarkers and methods for FDA-approved, MET-targeted drugs in NSCLC [59].

Publication	Drug	Method	Biomarker	N	ORR%
Moro-Sibilot et al. [39]	Crizotinib	FISH	*MET* GCN ≥ 6	25	16
		NGS	*MET* exon 14 skip	25	12
Landi et al. [40]	Crizotinib	FISH	*MET/*CEP7 > 2.2	16	31
		NGS	*MET* exon 14 skip	10	20
Drilon et al. [41]	Crizotinib	NGS	*MET* exon 14 skip	65	32
Schuler et al. [50]	Capmatinib	FISH	*MET* GCN < 4	17	6
			*MET* GCN 4–6	12	25
			*MET* GCN ≥ 6	15	47
			*MET/*CEP7 > 2.0	9	44
			*MET/*CEP7 < 2.0	32	22
		IHC	MET IHC 2+	14	14
			MET IHC 3_	37	27
Wu et al. [51]	Capmatinib with gefitinib	FISH	*MET* GCN < 4	41	12
			*MET* GCN 4–6	18	22
			*MET* GCN ≥ 6	36	47
		IHC	MET IHC 2+	16	19
			MET IHC 3+_	37	27
Wolf et al. [6]	Capmatinib	NGS	*MET* exon 14 skip (Previously treated)	69	41
			*MET* exon 14 skip (Untreated)	28	64
		NGS	*MET* GCN < 4 (Previously treated)	30	7
			*MET* GCN 4–5 (Previously treated)	54	9
			*MET* GCN > 6–9 (Previously treated)	42	12
			*MET* GCN ≥ 10 (Previously treated)	69	28
			*MET* GCN ≥ 10 (Untreated)	15	40
Paik et al. [31]	Tepotinib	NGS	*MET* exon 14 skip	99	46

**Table 5:** Adverse events in all cohorts (n = 364) in the GEOMETRY mono-1 trial [6].

Adverse Event	Total	Grade 3 or 4
Any event—No. (%)	355 (98)	244 (67)
Most common events—No. (%)		
Peripheral edema	186 (51)	33 (9)
Nausea	163 (45)	9 (2)
Vomiting	102 (28)	9 (2)
Blood creatinine increased	89 (24)	0
Dyspnea	84 (23)	24 (7)
Fatigue	80 (22)	16 (4)
Decreased appetite	76 (21)	3 (1)
Constipation	66 (18)	3 (1)
Diarrhea	64 (18)	2 (1)
Cough	58 (16)	2 (1)
Back Pain	54 (15)	3 (1)
Pyrexia	50 (14)	3 (1)
ALT increased	48 (13)	23 (6)
Asthenia	42 (12)	13 (4)
Pneumonia	39 (11)	17 (5)
Weight loss	36 (10)	2 (1)
Noncardiac chest pain	35 (10)	4 (1)
Serious adverse event—No. (%)	184 (51)	152 (42)
Event leading to discontinuation—No. (%)	56 (15)	35 (10)

**Table 6:** Current key ongoing studies involving capmatinib.

Clinical Trial Number	Phase	Purpose
NCT04427072	Phase III	Previously treated advanced NSCLC patients with *MET* exon 14 skipping mutation treated with capmatinib versus docetaxel
NCT04926831	Phase II	Efficacy and safety of neoadjuvant and adjuvant capmatinib
NCT05435846	Phase I/Ib	Capmatinib plus trametinib in patients with *MET* exon 14 skipping mutation
NCT04677595	Phase II	Chinese patients who are *EGFR* wt and *ALK* rearrangement negative with *MET* exon 14 skipping mutation
NCT05110196	Phase IV	Indian patients with *MET* exon 14 skipping mutation
NCT05488314	Phase I/II	Combination therapy of capmatinib and amivantamab in unresectable Stage IV NSCLC in patients with *MET* exon 14 skipping mutations or *MET* amplification
NCT05642572	Phase II	Combination therapy of capmatinib with osimertinib +/− ramucirumab in *EGFR* mutant, *MET*-amplified, Stage IV or recurrent NSCLC

## Abbreviations

NSCLC	Non-small cell lung cancer
EGFR	Epidermal growth factor receptor
KRAS	Kirsten rat sarcoma virus
BRAF	v-raf murine sarcoma viral oncogene homolog B1
ALK	Anaplastic lymphoma kinase
ROS1	Proto-oncogene tyrosine–protein kinase ROS
RET	Rearranged during transfection proto-oncogene
MET	Mesenchymal–epithelial transition
ERBB2	erb-b2 receptor tyrosine kinase 2
NTRK	Neurotrophic tyrosine receptor kinase
HGF	Hepatocyte growth factor
AMPK	AMP-activated protein kinase
LKB1	Liver kinase B1
GCN	Gain of copy number
FDA	U.S. Food and Drug Administration
EMA	European Medicines Agency
po	Oral
DLT	Drug limiting toxicity
b.i.d.	Twice a day
R2PD	Recommended Phase II dose
ORR	Overall response rate
PFS	Progression-free survival
DOR	Duration of response
IO	Immunotherapy
IHC	Immunohistochemistry
CEP7	Chromosome 7 centromere
ctDNA	Circulating tumor DNA
cfDNA	Circulating-free DNA
CTCs	Circulating tumor cells
FFPE	Fresh-frozen paraffin-embedded
PPA	Positive percent agreement
NPA	Negative percent agreement
OA	Overall agreement
NCCN	National Comprehensive Cancer Network
Teliso-V	Telisotuzumab vedotin
